# Immune responses during COVID-19 breakthrough cases in vaccinated children and adolescents

**DOI:** 10.3389/fimmu.2024.1372193

**Published:** 2024-05-15

**Authors:** Daniela Rivera-Pérez, Constanza Méndez, Benjamín Diethelm-Varela, Felipe Melo-González, Yaneisi Vázquez, Xing Meng, Qianqian Xin, Rodrigo A. Fasce, Jorge Fernández, Judith Mora, Eugenio Ramirez, Mónica L. Acevedo, Fernando Valiente-Echeverría, Ricardo Soto-Rifo, Alba Grifoni, Daniela Weiskopf, Alessandro Sette, Patricio Astudillo, Nicole Le Corre, Katia Abarca, Cecilia Perret, Pablo A. González, Jorge A. Soto, Susan M. Bueno, Alexis M. Kalergis

**Affiliations:** ^1^ Millennium Institute on Immunology and Immunotherapy, Santiago, Chile; ^2^ Facultad de Ciencias Biológicas, Pontificia Universidad Católica de Chile, Santiago, Chile; ^3^ Departamento de Ciencias Biológicas, Facultad de Ciencias de la Vida, Universidad Andrés Bello, Santiago, Chile; ^4^ Sinovac Biotech, Beijing, China; ^5^ Departamento de Laboratorio Biomédico, Instituto de Salud Pública de Chile, Santiago, Chile; ^6^ Laboratorio de Virología Molecular y Celular, Programa de Virología, Instituto de Ciencias Biomédicas, Facultad de Medicina, Universidad de Chile, Santiago, Chile; ^7^ Center for Vaccine Innovation, La Jolla Institute for Immunology (LJI), La Jolla, CA, United States; ^8^ Department of Medicine, Division of Infectious Diseases and Global Public Health, University of California San Diego (UCSD), La Jolla, CA, United States; ^9^ Departamento de Enfermedades Infecciosas e Inmunología Pediátrica, División de Pediatría, Escuela de Medicina, Pontificia Universidad Católica de Chile, Santiago, Chile; ^10^ Departamento de Endocrinología, Facultad de Medicina, Pontificia Universidad Católica de Chile, Santiago, Chile

**Keywords:** inactivated SARS-CoV-2 vaccine, CoronaVac^®^, pediatric, phase 3 clinical trial, omicron variant, breakthrough cases, SARS-CoV-2

## Abstract

**Background:**

Vaccine effectiveness against SARS-CoV-2 infection has been somewhat limited due to the widespread dissemination of the Omicron variant, its subvariants, and the immune response dynamics of the naturally infected with the virus.

**Methods:**

Twelve subjects between 3-17 years old (yo), vaccinated with two doses of CoronaVac^®^, were followed and diagnosed as breakthrough cases starting 14 days after receiving the second dose. Total IgGs against different SARS-CoV-2 proteins and the neutralizing capacity of these antibodies after infection were measured in plasma. The activation of CD4^+^ and CD8^+^ T cells was evaluated in peripheral blood mononuclear cells stimulated with peptides derived from the proteins from the wild-type (WT) virus and Omicron subvariants by flow cytometry, as well as different cytokines secretion by a Multiplex assay.

**Results:**

2 to 8 weeks post-infection, compared to 4 weeks after 2^nd^ dose of vaccine, there was a 146.5-fold increase in neutralizing antibody titers against Omicron and a 38.7-fold increase against WT SARS-CoV-2. Subjects showed an increase in total IgG levels against the S1, N, M, and NSP8 proteins of the WT virus. Activated CD4^+^ T cells showed a significant increase in response to the BA.2 subvariant (p<0.001). Finally, the secretion of IL-2 and IFN-γ cytokines showed a discreet decrease trend after infection in some subjects.

**Conclusion:**

SARS-CoV-2 infection in the pediatric population vaccinated with an inactivated SARS-CoV-2 vaccine produced an increase in neutralizing antibodies against Omicron and increased specific IgG antibodies for different SARS-CoV-2 proteins. CD4^+^ T cell activation was also increased, suggesting a conserved cellular response against the Omicron subvariants, whereas Th1-type cytokine secretion tended to decrease.

**Clinical Trial Registration:**

clinicaltrials.gov #NCT04992260

## Introduction

The severe acute respiratory syndrome coronavirus 2 (SARS-CoV-2) was responsible for the coronavirus disease-2019 (COVID-19) pandemic (1), which lasted more than three years. According to the World Health Organization (WHO), since its emergence in 2019, SARS-CoV-2 infections have caused at least 6.95 million deaths worldwide (2, 3). In response, an accelerated and successful global effort of developing different vaccines was carried out and shown to protect the population against the severe diseases caused by this virus (4). The pediatric population showed lower SARS-CoV-2 infection rates than the adult population (5). Nevertheless, the symptomatology of SARS-CoV-2 infection in the pediatric population is usually mild to moderate. However, in some cases, it manifests as a severe respiratory disease requiring admission to the pediatric intensive care unit (6). Severe post-infection complications have been reported in this context, such as pediatric multisystem inflammatory syndrome (MIS-C) (7, 8).

Vaccines based on viral mRNA, recombinant proteins, and inactivated SARS-CoV-2 virus have been approved for use in children and adolescents (9). These vaccines have shown to be safe, effective, and highly immunogenic due to the induction of a robust neutralizing antibody response against the ancestral virus (wild-type) in most evaluated pediatric cohorts (10–12). However, the emergence of the Omicron variant and its descendant subvariants (13) has diminished the efficacy of these vaccines against infection due to the limited neutralizing response induced by the ancestral virus-based vaccines (12). A similar response has been observed in convalescent children, who did not display effective antibodies against this variant (14). Such a scenario can increase the risk of breakthrough cases (14) and reinfections (15).

CoronaVac^®^ is an inactivated virus vaccine against SARS-CoV-2, which is safe and displays a protective immunogenicity profile with high efficacy against severe disease in adult (16–18) and pediatric (11) populations. Previous reports from our laboratory showed that two doses of CoronaVac^®^ in a 0-28 days schedule were capable of inducing secretion of neutralizing antibodies and the activation of CD4^+^ T cells at 4 weeks after the 2^nd^ dose in children and adolescents from 3 to 17 years. However, a reduced response against the Omicron variant was found (19). Here, we evaluated the clinical history and immune response of twelve subjects enrolled in an Open-label, phase III trial to test an inactivated SARS-CoV-2 vaccine (CoronaVac^®^) in the pediatric population, who were reported as breakthrough cases between February and June 2022 (19).

## Materials and methods

### Study design and subjects

The open-label study conducted in Chile (PedCoronaVac03CL), derived from “a multi-center international phase III clinical trial (clinicaltrials.gov #NCT04992260)”, evaluated the safety and immunogenicity of the inactivated SARS-CoV-2 vaccine CoronaVac^®^ in children and adolescents (19). The diagnosis and follow-up of subjects diagnosed with COVID-19 were carried out only in the center that evaluated immunogenicity. The study included a cohort of children and adolescents aged 3 to 17 years, distributed by age ranges of 3-5, 6-11, and 12-17 years old (yo), recruited from eleven different sites. The subjects were inoculated with two doses of 3 µg (600 standard units) of CoronaVac^®^ in a 0-28 schedule (two doses separated by 28 days or 4 weeks) considering a complete list of inclusion and exclusion criteria, which were previously published (19).

### Breakthrough cases

Positive COVID-19 cases (breakthrough cases) were defined as subjects with respiratory symptoms confirmed by a quantitative real-time-polymerase chain reaction (RT-qPCR) test. Each fully vaccinated subject evaluated in this study was notified with a positive diagnosis of SARS-CoV-2 between February and June 2022. All subjects whose diagnosis occurred more than 14 days after the second dose were included. To identify COVID-19 cases, parents or legal representatives were instructed to report through an electronic platform such as e-mail, text message, or telephone call each time the definition of a suspected positive case was met. A positive case was suspected if at least one of the following symptoms was present for more than two days: fever, cough, anosmia, ageusia, running nose, vomiting, or headache. Three days after symptom onset, an appointment with a physician was scheduled, and a nasopharyngeal swab or saliva sample was collected to assess the presence of SARS-CoV-2 RNA by RT-qPCR. Personnel of the study center followed the clinical evolution of the case until its resolution. No hospitalization was required for any of the breakthrough subjects.

### Sample collection

**Figure 1:**
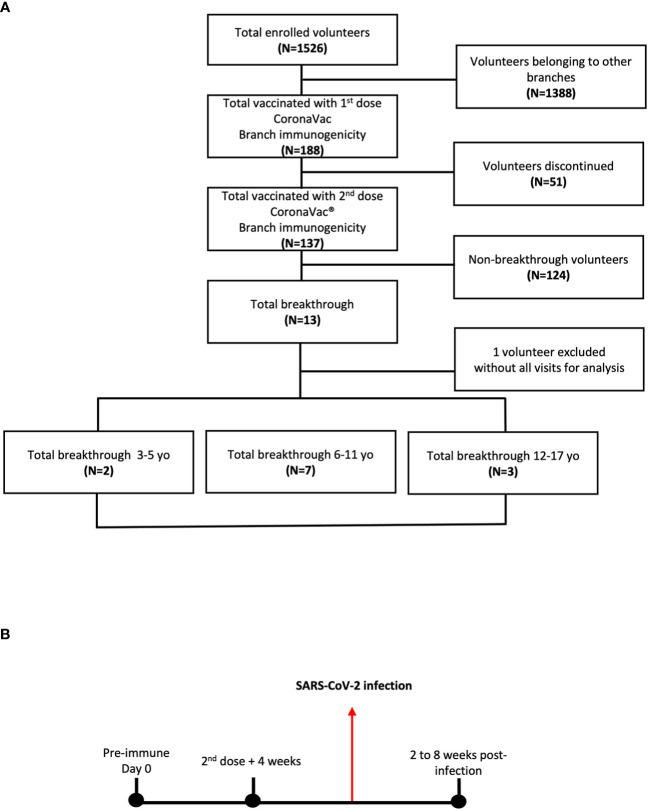
Immunogenicity branch cohort of study volunteers and their breakthrough cases of children and adolescents vaccinated with CoronaVac^®^. **(A)** A cohort of 188 volunteers receiving the first dose of CoronaVac^®^ was enrolled. Of the 188 volunteers, 137 received the second dose in a 0-28 vaccination schedule. 12 volunteers diagnosed with COVID-19 after the second dose + 4 weeks were analyzed as the study progress. **(B)** Blood samples were obtained at the time of the first vaccine dose on the pre-immune or day 0, at the time of the 2^nd^ dose + 4 weeks and 2 to 8 weeks after obtaining the positive diagnosis by qPCR for COVID-19.

Peripheral blood samples were collected in heparin-coated tubes during visits conducted at three different times: before administration of the first dose of the vaccine (pre-immune), 4 weeks after the 2^nd^ dose (2^nd^ dose + 4 weeks), and 2 to 8 weeks after confirmation of a positive COVID-19 case (post-infection) (**Figure 1B**). Although the timing of vaccine-related sample collection was the same among subjects, the collection of samples associated with infection differed over time because it depended on the time each subject was exposed to the virus. The samples were processed as previously reported (16, 19) to obtain plasma and peripheral blood mononuclear cells (PBMCs) for measuring humoral and cellular immunity, respectively.

### Evaluation of total IgG and neutralizing antibodies

Total IgG titers for the structural proteins Spike (S1: subunit 1) (SinoBiological, #40591-V08B1), Nucleocapsid (N) (R&D systems, #10474-CV), Membrane (M) (R&D systems, #10690-CV), and Envelope (E) (SinoBiological, #40609-V10E3), as well as the Non-Structural Protein 8 (NSP8) (R&D systems, #10633-CV) of SARS-CoV-2, were tested by in-house indirect ELISA assays, as previously described (20). The methodology is detailed in the **Supplementary Methods** section.

To determine the neutralizing capacity of the antibodies, three different assays were performed: surrogate virus neutralization test (sVNT) (21), pseudotyped neutralization test (pVNT) (21), and conventional virus neutralization test (cVNT) (21). The methodology is detailed in the **Supplementary Methods** section.

### Activation of CD4^+^ and CD8^+^ T cell populations using SARS-CoV-2-derived peptide megapools of SARS-CoV-2

Activation-induced markers (AIM^+^) CD4^+^ and CD8^+^ T cells were evaluated in stimulated PBMCs purified from the 12 breakthrough cases by flow cytometry. The following MP of peptides derived from SARS-CoV-2 proteins were obtained from the La Jolla Institute of Immunology and used for the subsequent stimulus: MP-S WT (Spike protein of the wild-type strain) and MP-R (viral proteins excluding Spike) (19, 22). Furthermore, two specific commercial MPs against S protein of BA.1 and BA.2 Omicron subvariants were included. For MPs R and S, all 12 subjects were evaluated. However, for the BA.1 and BA.2 MPs, 11 of the 12 subjects were assessed because no cells were available for one of them (subject 1 was not included in this assay). A concentration of 0.6 nM of each MP was used for the subvariants (Miltenyi, Cod: BA.1 #130-129-928, BA.1 WT reference #130-129-927, BA.2 #130-128- 763, reference BA.2 WT #130-128-761). As a positive control, 1.62 mM/0.6 mM PMA (Sigma Aldrich, code no. P1585) and ionomycin (Sigma Aldrich, code no. 3909) were used and 1% DMSO (N. Merck No. 317275) as a negative control. The methodology is detailed in the **Supplementary Methods** section.

### Multiplex assays for cytokine determination

Supernatants from PBMCs stimulated with SARS-CoV-2 MPs (from the cytometry assay) for 24h were evaluated using the Multiplex assay technology (R&D systems, #LXSAHM-04, USA) to assess interleukin (IL)-2, interferon-gamma (IFN-γ), IL-4 and IL-17 production. IL-2 and IFN-γ were evaluated as they promote a Th1 response, characteristic of the protective antiviral response. On the other hand, it is suggested that IL-4 and IL-17 are associated with a pro-inflammatory response and viral persistence (22). The supernatants of samples stored at -80°C were thawed at room temperature. After 2h incubation of supernatants with magnetic beads coated with analyte-specific primary antibodies, the samples were incubated with analyte-specific secondary antibodies conjugated with biotin. Then, the samples were incubated with streptavidin R-phycoerythrin and analyzed using a Luminex^®^ 200 xMAP multiplex system (Luminex Corporation, Austin, TX). According to the manufacturer’s instructions, the detection limit for the cytokines measured ranged from 40 to 9,800 pg/mL.

### Ethical considerations

This study was approved by the institutional Scientific Ethical Committee of Health Sciences at the Pontificia Universidad Católica de Chile (#210616012) and by the Instituto de Salud Pública de Chile (#20674/21). It was conducted according to the current Tripartite Guidelines for Good Clinical Practices, the Declaration of Helsinki (23), and local regulations.

### Statistical analyses

Statistical analysis of total antibodies, measured as a geometric mean titer (GMT) and geometric mean units (GMU), expressed in BAU/mL for anti-S1, anti-N, and anti-M antibodies), neutralizing antibodies (measured as GMT), T cell populations (measured as %AIM^+^ cells), and cytokine concentrations (measured as pg/mL) was performed employing mixed effects models, where the variables (1) time from the pre-immune visit, and (2) subject visit (identified as pre-immune, 2^nd^ dose + 4 weeks, and 2 to 8 weeks post-infection) were set as fixed effects, and the individual subjects (identified through unique subject ID codes) were set as random effects. These models allowed for assessing the impact of the passage of time since the pre-immune visit and the difference among visits in the different response variables. Statistical modeling, testing, and figure construction were performed using the R programming language version 4.2.2 (24).

## Results

### Subject recruitment and clinical features of breakthrough cases

A total of 188 participants were enrolled in the center for the immunogenicity branch under a 0-28 immunization schedule. From the initial group, 137 subjects received both doses of CoronaVac^®^. After the administration of the second dose of CoronaVac^®^, 13 subjects were diagnosed as COVID-19 positive by RT-qPCR for SARS-CoV-2 and reported as breakthrough cases in the period from February to June 2022 (3-5 yo: 3 individuals, 6-11 yo: 7 individuals, and 12-17 yo: 3 individuals). One subject was excluded due to the absence of samples at different time points; therefore, twelve subjects were finally included in this study (**Figure 1A**). For the immunogenicity analyses, blood samples were obtained at three different times (pre-immune, 4 weeks after 2^nd^ dose, and 2 to 8 weeks post-infection) (**Figure 1B**). As indicated in the Methods section, the visits for the “4 weeks post 2^nd^ dose” and “2 to 8 weeks post-infection” occurred at variable time points relative to the pre-immune visit for the different subjects. In particular, the “2 to 8 weeks post-infection” visit occurred at a broad interval of times relative to the pre-immune visit (**Figure 1B**). The highest prevalence of COVID-19-positive cases (breakthrough cases) was recorded in the 6 to 11 yo age group, with 58.3% (**Table 1**). Then, the group aged 12 to 17 yo presented a prevalence of 25% and the 3-5 yo group of 16.7%. All cases were considered mild, with a mean duration of symptoms in all groups of 7.5 ± 3.8 days (**Table 1**). The most common clinical signs reported were fever and runny nose in 75% (9 subjects), cough in 66.6% (8), and headache in 58.3% (7). Only one case showed vomiting (8.3%), and none showed anosmia or ageusia. None of the subjects required hospitalization (**Table 1**). Six subjects (50%) of the 12 breakthrough cases had comorbidities when enrolled. The most frequent comorbidity was allergic rhinitis in 25% of the cases. Asthma, mood disorder, anxiety, precocious puberty, hypermenorrhea, and obesity only had a prevalence of 8.3% (**Table 1**). The specific comorbidities are shown for each subject in the **Supplementary Table 2**.

**Table 1:** Demographics and clinical features of breakthrough cases.

Item	Total breakthrough cases of the pediatric study (n=12)	Percentage (%)
**Time symptoms** (mean days± SD)	7.5 ± 3.8	Not applicable
Age range		
3-5 yo	2	16.7
6-11 yo	7	58.3
12-17 yo	3	25.0
Sex		
Female	7	58.3
Male	5	41.7
Signs/Symptoms		
Fever	9	75.0
Runny nose	9	75.0
Cough	8	66.6
Headache	7	58.3
Vomit	1	8.3
Anosmia	0	0
Ageusia	0	0
Comorbidities		
Present/Absent	6/6	50.0
Allergic rhinitis	3	25.0
Asthma	1	8.3
Early puberty	1	8.3
Mood disorder	1	8.3
Anxiety	1	8.3
Hypermenorrhea	1	8.3
Obesity	1	8.3
Severity		
Mild	12	100
Moderate	0	0
Serious	0	0
Required hospitalization	0	0

SD, standard deviation; yo, years-old.

### Humoral immune response against SARS-CoV-2 in breakthrough cases

**Figure 2:**
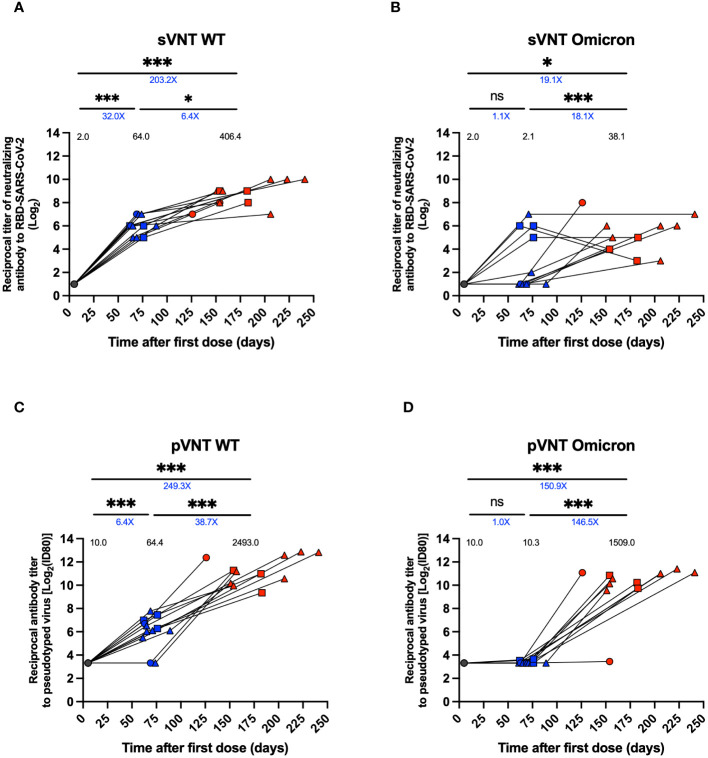
Titers of neutralizing antibodies against SARS-CoV-2 in plasma of breakthrough cases of children and adolescents. Titers of neutralizing antibodies against SARS-CoV-2 in twelve breakthrough cases. **(A, B)** Neutralizing experiment titers evaluated by a surrogate viral neutralization test (sVNT) expressed as geometric mean titer (GMTs) for WT virus and Omicron variant, respectively. **(C, D)** Neutralizing experiment titers evaluated by pseudotyped based viral neutralization assay (pVNT) with ID80 expressed as GMTs for WT virus and Omicron variant, respectively. Circles, triangles and squares correspond to subjects 3-5 yo, 6-11 yo and 12-17 yo, respectively. On the other hand, the black, blue and red colors represent the samples evaluated at pre-immune, 2^nd^ dose + 4 weeks and 2 to 8 weeks post-infection, respectively. The values on each column indicate the geometric mean. The values below the significance line indicate the fold-change between the corresponding geometric means titers (Red: decrease, Blue: increase). Transformed data, represented as reciprocal dilution in logarithm base 2 on linear scale. Data was analyzed using a mixed effects model, ***p<0.001; *p<0.05, not significant (ns).

Neutralizing antibody titers were assessed using sVNT, pVNT, and cVNT, as previously described (21). A 32-fold increase in neutralizing antibody titers for WT SARS-CoV-2 (GMT 64.0 vs. 2.0, p<0.001) and a 1.1-fold increase for the Omicron variant (2.1 vs. 2.0, ns) were observed in the 2^nd^ dose + 4 weeks samples relative to the pre-immune time (**Figures 2A, B**). On the other hand, a 6.4-fold increase in neutralizing titers for WT (GMT 406.4 vs. 64.0 p<0.05) and an 18.1-fold increase for the Omicron variant (38.1 vs. 2.1, p<0.001) was observed in the sample from 2 to 8 weeks post-infection relative to the 2^nd^ dose + 4 weeks in the sVNT analyses (**Figures 2A, B**). Equivalent results were obtained from pVNT assays, with a 38.7-fold increase after infection for WT (GMT 2493.0 vs. 64.4, p<0.001), and a 146.5-fold increase was observed in the samples from 2 to 8 weeks post-infection relative to the 2^nd^ dose + 4 weeks for Omicron (GMT 1509.0 vs. 10.3, p<0.001) (**Figures 2C, D**). Similarly, in the results obtained by cVNT for the WT SARS-CoV-2 virus, a moderate increase of neutralizing antibodies was observed from the 2^nd^ dose + 4 weeks to 2 to 8 weeks post-infection (**Supplementary Figure 1**). During pre-immune, 2^nd^ dose + 4 weeks and 2 to 8 weeks post-infection visits, seropositivity and seroconversion were 100% for the WT virus. However, seropositivity to the Omicron variant was 0% in 2^nd^ dose + 4 weeks but 100% in 2 to 8 weeks post-infection (**Supplementary Table 3**).

**Figure 3:**
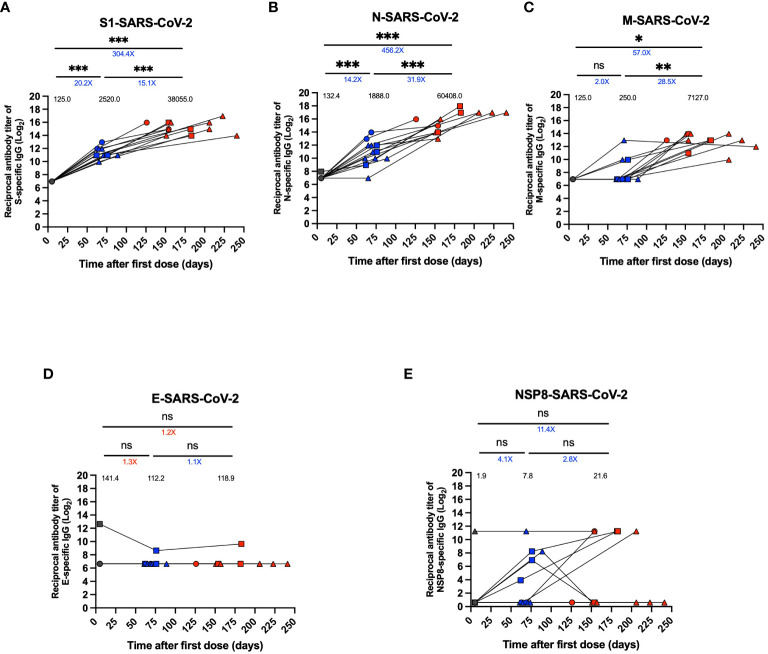
Specific IgG levels against different SARS-CoV-2 proteins in plasma of breakthrough cases of children and adolescents. Titers of specific IgG levels against different SARS-CoV-2 in twelve breakthrough cases. **(A)** Antibody titer against S1 protein. **(B)** Antibody titer against N protein. **(C)** Antibody titer against M protein. **(D)** Antibody titer against E protein. **(E)** Antibody titer against NSP8 protein. An *in-house* indirect ELISA assay was used to assess plasma IgG specific antibody titers against these proteins. Data were transformed, plotted as reciprocal dilution in log base 2 on a linear scale. Circles, triangles and squares correspond to subjects 3-5 yo, 6-11 yo and 12-17 yo, respectively. On the other hand, the black, blue and red colors represent the samples evaluated at pre-immune, 2^nd^ dose + 4 weeks and 2 to 8 weeks post-infection, respectively, by before-after graph. Black values below the significance line indicate geometric mean titers (GMT), blue values above the significance line indicate an increase in GMT of the two time points compared, and red values above the significance line indicate a decrease in GMT of the two time points compared. The data was analyzed using a mixed effects model, ***p<0.001; **p<0.01; *p<0.05, not significant (ns).

Specific IgG antibodies were quantified for structural proteins S1, N, M, and E and the non-structural NSP8 protein of SARS-CoV-2. For the anti-S1 response, a 20.2-fold increase in titers per GMT and an 8.4-fold increase per GMU was observed at the 2^nd^ dose + 4 weeks compared to the pre-immune time (GMT 2520.0 vs. 125.0, p<0.001, GMU 869.1 vs. 103.3, p <0.001) (**Figure 3A**; **Supplementary Figure 2A**). On the other hand, there was a 15.1-fold increase in the 2 to 8 weeks post-infection time and a 27.2-fold increase per GMU compared to the 2^nd^ dose + 4 weeks visit (GMT 38055.0 vs. 2520.0, p<0.001; GMU 23624 vs. 869.1, p<0.001) (**Figure 3A**; **Supplementary Figure 2A**).

The anti-N response showed a 14.2-fold increase in antibody titers at the time of the 2^nd^ dose + 4 weeks per GMT and a 4.4-fold increase per GMU compared to pre-immune time (GMT 1888.0 vs. 132.4, p<0.001; GMU 72.0 vs. 16.3, p<0.001) (**Figure 3B**, **Supplementary Figure 2B**). After infection, there was a 31.9-fold increase in antibody titers per GMT and an 18.1-fold increase per GMU compared to the time after the second dose and before infection (GMT 60408.0 vs. 1888.0, p=0.001; GMU 1303.0 vs. 72.0, p<0.001) (**Figure 3B**; **Supplementary Figure 2B**).

In contrast, for the anti-M response, there was only a significant 28.5-fold increase in the antibody titers from the second dose + 4 weeks visit to 2 to 8 weeks post-infection time for GMT and a non-significant increase for GMU (GMT 7127.0 vs. 250.0, p<0.01, GMU 223.7 vs. 80.3, p>0.05) (**Figure 3C**; **Supplementary Figure 2C**). Finally, there was a post-infection of 57.0-fold increase compared to pre-immune in GMT, and a 4.0-fold increase moderate for GMU (GMT 7127.0 vs. 125.0, p<0.05; GMU 223.7 vs. 56.3, p>0.05) (**Figure 3C**; **Supplementary Figure 2C**). In addition, for the anti-E and anti-NSP8 responses, no significant changes were observed during the evaluated period (**Figures 3D, E**), except for one subject that showed elevated anti-E antibody titers at all visits (**Figure 3D**). Additionally, for NSP8, one subject had elevated anti-NSP8 antibody titers at the pre-immunization visit that were maintained through the post-infection visit, and only some subjects showed moderately increased titers after the second dose + 4 weeks and an increase in titers after infection, but it was not significant (**Figure 3E**). Seropositivity and seroconversion for the different virus proteins were evaluated for samples obtained during the 2^nd^ dose + 4 weeks visit and the 2 to 8 weeks post-infection visit. S1 protein-specific antibodies were elevated in 100% of the samples in all the periods evaluated (**Supplementary Table 4**). In contrast, antibody positivity was greater than 90% pre-infection for the N protein and reached 100% post-infection (**Supplementary Table 4**). However, the M protein showed only 33% seropositivity pre-infection but reached 100% post-infection (**Supplementary Table 4**). On the other hand, the E protein-specific antibodies showed a 0% seropositivity in 2^nd^ dose + 4 weeks and 2-8 weeks post-infection (**Supplementary Table 4**). NSP8 protein seropositivity decreased post-infection from 33.3% to 25.0% (**Supplementary Table 4**).

### CD4^+^ and CD8^+^ T cell activation upon stimulation with WT and Omicron SARS-CoV-2

**Figure 4:**
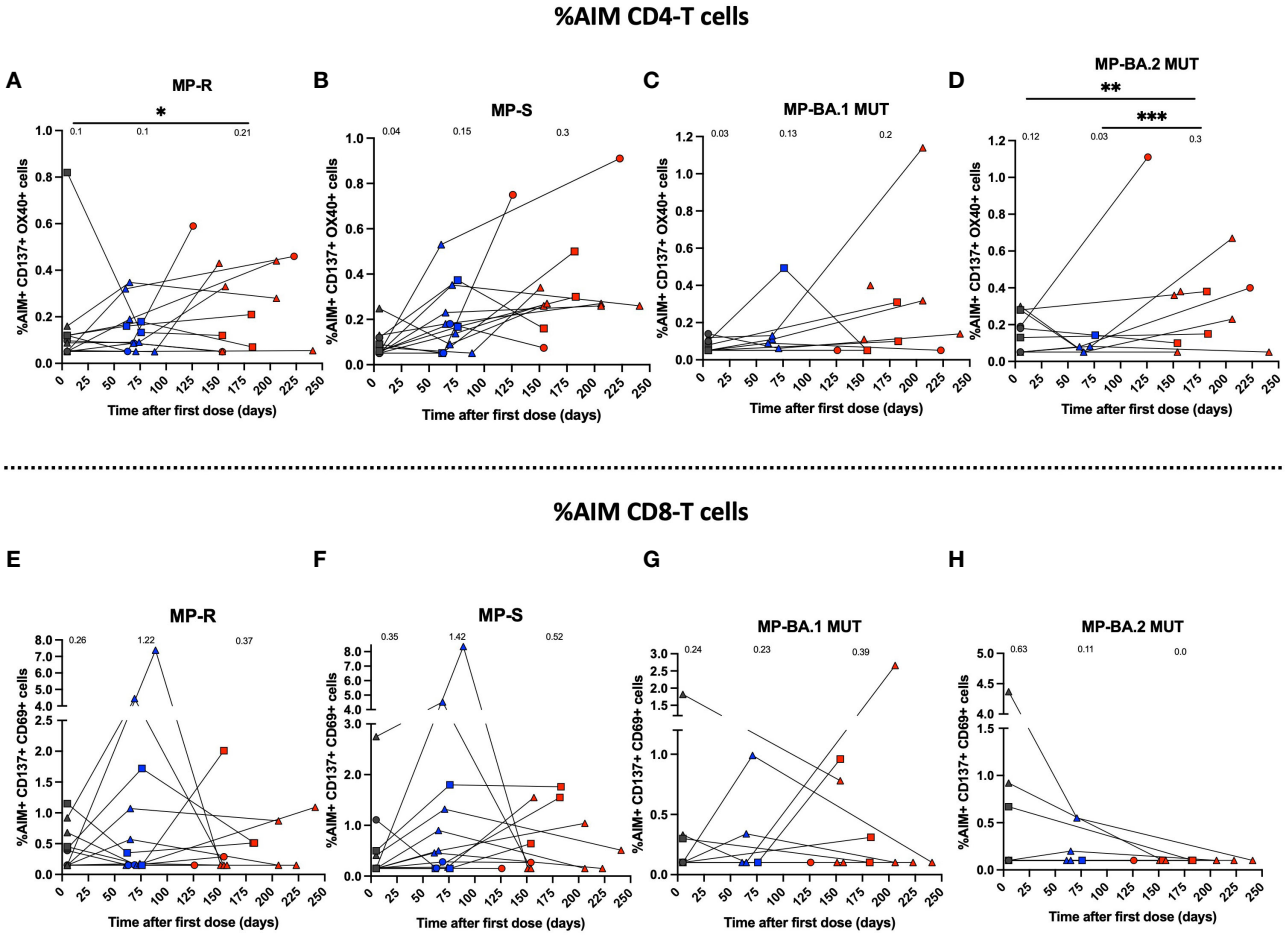
Immune response through activation of T cell populations using MPs of SARS-CoV-2 of breakthrough cases of children and adolescents. Cellular response in breakthrough pediatric cases was assessed in PBMCs. **(A–D)** Percentage (%) AIM^+^ CD4^+^ T for MPs R, S of WT SARS-CoV-2 and BA.1-MUT and BA.2-MUT subvariant Omicron MPs, respectively. **(E–H)**. Percentage (%) AIM^+^ CD8^+^ T for MPs R, S of WT SARS-CoV-2 and BA.1-MUT and BA.2-MUT subvariant Omicron MPs, respectively. The percentage of AIM^+^ CD4^+^T (CD137^+^OX40^+^) and AIM^+^ CD8^+^ T (CD137^+^CD69^+^) was determined by flow cytometry. Circles, triangles and squares correspond to subjects 3-5 yo, 6-11 yo and 12-17 yo, respectively. On the other hand, the black, blue and red colors represent the samples evaluated at pre-immune, 2^nd^ dose + 4 weeks and 2 to 8 weeks post-infection, respectively. The data was analyzed using a mixed effects model, ***p<0.001; **p<0.01; *p<0.05. The absence of * indicates that there is no statistical significance.

For cellular immunity analyses, PBMCs were stimulated with the peptide MP of SARS-CoV-2 R and S of the WT strain (**Figures 4A, B, E, F**). In addition, MPs against Omicron’s BA.1 and BA.2 subvariants were used with their respective reference MPs (**Figures 4C, D, G, H**). With these MP, activation (AIM^+^) of CD4^+^ and CD8^+^ T cells was assessed by flow cytometry. We observed a significant increase in activated SARS-CoV-2-specific CD4^+^ T cells at pre-immune and 2 to 8 weeks post-infection times for R (p<0.05) (**Figure 4A**). On the other hand, we observed an increase in the activation of CD4^+^ T cells for the S and BA.1 stimulus between the pre-immune visit and the 2^nd^ dose + 4 weeks, and between 2^nd^ dose + 4 weeks to 2 to 8 weeks post-infection visit, but this result was not statistically significant (**Figures 4B, C**). CD4^+^ T cell activation in response to the MP BA.2 subvariant was increased between pre-immune timepoint and 2 to 8 weeks post-infection (p<0.01) and between 2^nd^ dose + 4 weeks and 2 to 8 weeks post-infection (p<0.001) (**Figure 4D**). Subsequently, we assessed CD8^+^ T cell activation in response to SARS-CoV-2 antigens (**Figures 4E–H**). CD8^+^ T cell activation stimulated with MP-R, MP-S, and MP-BA.1 was increased for the 2^nd^ dose + 4 weeks and 4-8 weeks post-infection in some subjects; however, these differences were not statistically significant (**Figures 4E–G**). In contrast, stimulation with MP-BA.2 led to no significant increases in T cell activation at all evaluated times (**Figure 4H**).

### Cytokine in supernatants from PBMCs stimulated with SARS-CoV-2 antigens

**Figure 5:**
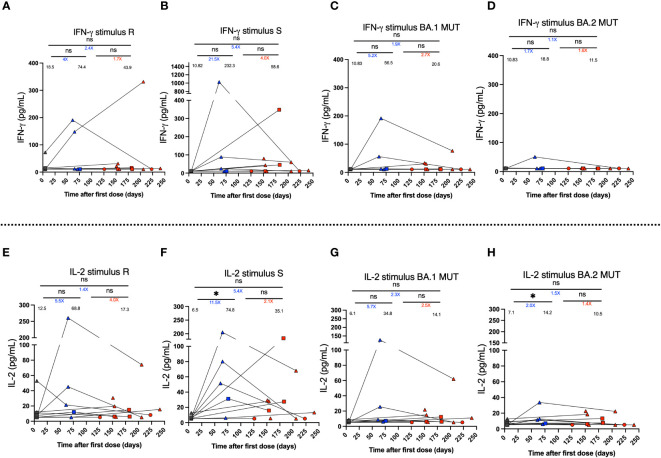
IFN-γ and IL-2 secretion by PBMCs stimulated using MPs of SARS-CoV-2 of breakthrough cases of children and adolescents vaccinated with CoronaVac^®^. IFN-γ and IL-2 secretion was quantified in supernatants of PBMCs of twelve breakthrough cases, upon stimulation with megapools of peptides derived from SARS-CoV-2 proteins by Multiplex assay. **(A–D)** IFN-γ secretion (pg/ml) by PBMCs stimulated with MP-R and MP-S of WT SARS-CoV-2, and MP-BA.1 and BA.2 of protein S of the Omicron variant of SARS-CoV-2, respectively. **(E–H)** IL-2 secretion (pg/ml). Circles, triangles and squares correspond to subjects 3-5 yo, 6-11 yo and 12-17 yo, respectively. On the other hand, the black, blue and red colors represent the samples evaluated at pre-immune, 2^nd^ dose + 4 weeks and 2 to 8 weeks post-infection, respectively, by before-after graph. The values below the significance line indicate the times of change between the corresponding media (Red: decrease, Blue: increase). The data was analyzed using a mixed effects model, *p<0.05, not significant (ns).

The secretion of the cytokines IFN-γ, IL-2, IL-4, and IL-17 was determined for PBMCs cultures stimulated during 24 h with SARS-CoV-2-derived MP peptides. Cytokine secretion was quantified for 11 vaccinated subjects in samples derived from the pre-immune, 2^nd^ dose + 4 weeks, and 2-8 weeks post-infection visits. However, not all of them were evaluated for all stimuli and times due to the limited number of cells obtained in these subjects. IFN-γ secretion had a mean increase after the second dose of CoronaVac^®^ for the R stimulus of 4-fold (**Figure 5A**) and S stimulus equal to 21.5-fold (**Figure 5B**), associated with the WT strain. In addition, BA.1 showed a 5.2-fold change (**Figure 5C**), and BA.2 showed a 1.7-fold change (**Figure 5D**) compared to the pre-immune samples, although this increase was not observed in all subjects. However, these results were not statistically significant (mean 74.4 vs. 18.5; 232.3 vs. 10.8; 56.5 vs. 10.8; 18.8 vs. 10.8, respectively). On the contrary, after infection, a non-significant decrease in IFN-γ secretion was observed for all stimuli (**Figures 5A–D**). For the R stimulus, a 1.7-fold decrease was observed (mean 74.4 vs. 43.9), and for the S stimulus, a 4-fold decrease in secretion was found (mean 232.3 vs. 58.6). For the stimuli associated with the Omicron BA.1 and BA.2 subvariants, the decrease was 2.7 and 1.6-fold, respectively (**Figures 5C, D**). The results of IL-2 secretion were the same as those observed for IFN-γ (**Figures 5E–H**). A significant increase (p<0.05) in IL-2 secretion was observed for MP-S (**Figure 5F**), although this peak was due to one subject. A non-significant post-SARS-CoV-2 infection decrease was observed for MPs R, S, BA.1, and BA.2 of 4.0-fold, 2.1-fold, 2.5-fold, and 1.4-fold, respectively. Also, the secretion of IL-4 and IL-17 was evaluated for the same subjects, however, the secretion levels were lower compared to IFN-γ and IL-2, but not significant. Specifically, the PBMCs did not secrete IL-17 at any of the times analyzed, and there was only one subject who, in the sample of the 2^nd^ dose + 4 weeks, presented an increase in the secretion of this cytokine compared to the pre-immune sample (**Supplementary Figure 3**).

## Discussion

Our study is the first to provide information on the immune response in breakthrough pediatric cases immunized with CoronaVac^®^. In this report, we provide the clinical history and immune response measured in 12 pediatric subjects (3-5 years: 2 subjects, 6-11 years: 7 subjects, and 12-17 years: 3 subjects) vaccinated with two doses of CoronaVac^®^, who became infected after the second dose. In breakthrough subjects, there was an increase in neutralizing antibody titers against Omicron after infection. In addition, CoronaVac^®^ vaccination generated specific IgG antibodies against other SARS-CoV-2 structural and non-structural proteins that increase post-infection. Regarding cellular immunity, there were no differences between the WT virus and the subvariants of Omicron BA.1 and BA.2, a factor that may help explain the enduring effectiveness of this vaccine against severe disease despite the emergence of SARS-CoV-2 variants with significant neutralizing antibody evasion properties (25). The activation of CD4^+^ T cells did not change post-infection compared to pre-infection.

In our study, of the 12 breakthrough cases, 6 (50%) had comorbidities before vaccination. Allergic rhinitis, asthma, and endocrine disorders in children and adolescents with or without these comorbidities display a similar risk of SARS-CoV-2 infection and severity (26–28), as observed in our study. On the other hand, no studies in the pediatric population indicate susceptibility and severity of COVID-19 related to comorbidities of mood disorders and anxiety, but it displayed an increased risk of infection, hospitalization, and death in the adult population (29–31). In contrast, obesity has been reported as a predisposing factor in the pediatric COVID-19-positive population (30). These conditions are associated with increased severity of infection (32, 33). However, in our study, subjects with these comorbidities did not present severe disease, consistent with results in adults vaccinated with CoronaVac^®^ (34, 35).

We evaluated the neutralizing capacity of antibodies against the Omicron variant compared to the WT strain using sVNT and pVNT. Consistent with other reports using other vaccine platforms, our results show that a second dose of CoronaVac^®^ (pre-infection sample) failed to increase neutralization titers against Omicron but did against WT virus (30). In contrast, we observed that SARS-CoV-2 infection after the second dose of CoronaVac^®^ in the pediatric population significantly increased the neutralization response against Omicron by both assays. These results are consistent with evidence from other studies in children immunized with BNT162b2 or Ad26.CoV2-S (32) and adults vaccinated with CoronaVac^®^ (33, 36) whom subsequently became infected, and infection was observed to improve the antibody neutralization response against Omicron. On the other hand, it has been reported that infection by Omicron and its subvariants BA.2.12.1, BA.4, and BA.5 promotes an increase in neutralizing antibodies in adults vaccinated with the mRNA platforms (BNT162b or mRNA-1273) against other variants of concern (37, 38), that could be long-lasting (39). In this regard, we observed a 5-fold increase in neutralizing antibody titers against the Delta variant after infection relative to pre-infection (results not shown).

To complement our results, we evaluated the induction profile of specific IgG-binding antibodies against the proteins S1, N, M, E and NSP8 of SARS-CoV-2 strain WT, because the CoronaVac^®^ vaccine formulation contains the complete inactivated viral particle, including all viral antigens, which is different to other vaccine platforms that contain only one viral antigen. The second dose of immunization (pre-infection sample) and subsequent infection promoted a significant increase in IgG antibodies specific against S1 and N proteins. In contrast, IgG anti-M increased only significantly post-infection. For the E protein, there was no response, except in a single subject, probably associated with cross-reactivity in IgG antibodies against the E protein of SARS-CoV-2 and other human coronaviruses (40). For the NSP8 protein, there was an increase, which was not significant, in pre-infection and post-infection states in some subjects. The IgG antibody response has been poorly reported in the pediatric population in the context of vaccination and infection. However, our results are consistent with what has been observed in adult breakthrough cases (20, 41). Noteworthy, the subjects herein only presented a significant increase in IgG anti-M titers post-infection and showed a tendency to present an improved response in pre-infection against the S1 and N proteins compared to what was reported in adults (19). Our results suggest that our vaccine mainly promotes responses against S1 and N proteins of the SARS-CoV-2 but not against M, E, and NSP8. This is the first report that evaluated these antigens in pediatric subjects in CoronaVac^®^ vaccinated and breakthrough cases. On the other hand, our results suggest the potential role of these antibodies against other antigens than S induced by vaccination with CoronaVac^®^ in the pediatric population.

Cellular immunity analysis evaluated the activation response of antigen-specific CD4^+^ T and CD8^+^ T cells in breakthrough cases by stimulation with WT SARS-CoV-2 protein S MPs and Omicron BA.1 and BA.2 MP subvariants. We observed a significant activation response of CD4^+^ T cells against MP-R and MP-BA.2 at the 2^nd^ dose + 4 weeks and 2 to 8 weeks post-infection visit, compared to the pre-immune visit. In contrast, particularly, the responses of the CD8^+^ T cells tended to display non-significant changes in activation against the different stimuli. These results suggest that subjects with SARS-CoV-2 infection could maintain the percentage of activated CD4^+^ T cells, avoiding its reduction in most evaluated subjects. We showed that this specific T-cell response against SARS-CoV-2 S protein was preserved against Omicron, as indicated in other studies (41) before infection, possibly accounting for enduring protection against severe and fatal disease regardless of the infecting virus variant (25). Our results were consistent with those observed in children (31) and adults vaccinated with mRNA or recombinant vector platforms, who were later infected with Omicron (42). Additionally, we observed increased %AIM for CD4^+^ T and CD8^+^ T cells stimulated with MP-R and MP-S at the pre-immune visit, where a cross-reactive response to other seasonal human coronaviruses has been described (39).

Finally, we assessed cytokine secretion to understand the SARS-CoV-2-associated immune response in pediatric breakthrough cases. The results showed a response that differs between subjects, where some achieve an increase in IFN-γ levels, and others maintain this cytokine at their basal level. Likewise, post-vaccination IL-2 levels differ between subjects. Although an increase in the means was observed for stimulation with S, the peak observed is due to one subject and did not represent the trend of all the samples. Previously, our research group showed a difference between the pre-immune condition and 2^nd^ dose + 4 weeks, specifically for the MP-R and MP-S stimuli in pediatric subjects (19). A larger number of subjects will likely allow for a significant trend in cytokine levels to be determined. Furthermore, in the MP-BA.1 and MP-BA.2 stimuli, the secretion was slightly less than in the other MPs. Particularly, after infection (2 to 8 weeks post-infection), the secretion of these cytokines tended to decrease but without significant changes. No investigations have been reported on the change in cytokine levels in pediatric breakthrough cases immunized with CoronaVac^®^, and our study is the first to report levels of cytokines in these volunteers. Other studies describe an increase in IFN-γ and IL-2 secretion in the unvaccinated pediatric population after symptomatic COVID-19 (41, 42), but a reduction in children with MIS-C (42), yet all our breakthrough cases presented mild COVID-19.

The main limitations of this study were the low number of reported cases and the low number of PBMCs obtained from subjects, which were not sufficient in all subjects for performing all cellular immunity and cytokine analyses.

In breakthrough pediatric cases vaccinated with two doses of CoronaVac^®^ in a 0-28 immunization schedule, an increase in neutralizing antibody levels against Omicron was observed after SARS-CoV-2 infection, which was maintained for at least 2 to 24 weeks post-infection. In addition, the infection promoted an increase in specific IgG antibodies against SARS-CoV-2 S1, N, M, and NSP8 proteins. On the other hand, activation of CD4^+^ T cells against S antigen and BA.1 Omicron subvariant was observed after vaccination and post-infection, accompanied by a non-significant decrease of CD8^+^ T cells post-infection for S, R antigen, and BA.2 subvariant.

We suggest that maintenance of the CD4^+^ T cell response post-infection may protect against future SARS-CoV-2 infections. Finally, after infection, the secretion of IFN-γ and IL-2 cytokines showed a discreet decrease trend after infection in some subjects, but this result is not statistically significant. Overall, the results presented here provide a novel clinical characterization of the immune response of the vaccinated pediatric population following breakthrough infections.

## Data availability statement

Data supporting the findings reported within the study are available from the corresponding authors upon reasonable request.

## Ethics statement

This study was approved by the institutional Scientific Ethical Committee of Health Sciences at the Pontificia Universidad Católica de Chile (#210616012) and by the Instituto de Salud Pública de Chile (#20674/21). The studies were conducted in accordance with the local legislation and institutional requirements. Written informed consent for participation in this study was provided by the participants’ legal guardians/next of kin.

## Author contributions

AMK: Conceptualization, Visualization, Methodology and Research, Funding Acquisition, Project Administration, Supervision, Writing – original draft, Writing – review & editing. SMB: Conceptualization, Visualization, Methodology and Research, Funding Acquisition, Project Administration, Supervision, Writing – original draft, Writing – review & editing. PAG: Conceptualization, Visualization, Methodology and Research, Funding Acquisition, Project Administration, Supervision, Writing – original draft, Writing – review & editing. XM: Conceptualization, Writing – review & editing. QX: Conceptualization, Writing – review & editing. JAS: Conceptualization, Visualization, Methodology and Research, Writing – review & editing. FM-G: Conceptualization, Visualization, Methodology and Research, Formal analysis, Writing – review & editing. CM: Conceptualization, Visualization, Methodology and Research, Formal analysis, Writing – original draft, Writing – review & editing. DR-P: Conceptualization, Visualization, Methodology and Research, Formal analysis, Writing – original draft, Writing – review & editing. BDV: Visualization, Methodology and Research, Formal analysis, Writing – review & editing. YV: Visualization, Methodology and Research, Writing – review & editing. KA: Methodology and Research, Writing – review & editing. RAF: Methodology and Research, Writing – review & editing. JF: Methodology and Research, Writing – review & editing. JM: Methodology and Research, Writing – review & editing. ER: Methodology and Research, Writing – review & editing. MLA: Methodology and Research, Writing – review & editing. FVE: Methodology and Research, Writing – review & editing. RSR: Methodology and Research, Writing – review & editing. AG: Methodology and Research, Writing – review & editing. AS: Methodology and Research, Writing – review & editing. DW: Methodology and Research, Writing – review & editing. NLC: Methodology and Research, Project Administration, Writing – review & editing. CP: Methodology and Research, Project Administration, Writing – review & editing. AK: Project Administration. PA: Writing – review & editing. PSG: Writing – review & editing. SB: Writing – original draft.
