# Supplementary material for: Immune responses during COVID-19 breakthrough cases in vaccinated children and adolescents
Source: Front Immunol. 2024 May 15;15:1372193. doi: 10.3389/fimmu.2024.1372193 (PMC11133585; doi:10.3389/fimmu.2024.1372193)
Supplement: Supplementary file 1 [file DataSheet_1.pdf]

Supplementary Methods Section

Evaluation of total IgG and neutralizing antibodies

The 96-well high-binding ELISA plates (Corning, #9018) were activated with 100 ng of S1, N, or M proteins dissolved in carbonate-bicarbonate buffer (Biolegend 1x, #421701) for 1 h at 37°C and blocked with 10% m/w milk in 1X PBS-Tween 20 (0.05%) overnight at 4°C. The E or NSP8 proteins were activated using 500 ng and 200 ng, respectively, dissolved in 20 mM monobasic and dibasic phosphate denaturing buffer, 0.5 NaCl, and 8 M urea for 1 h at 37°C and blocked with 10% m/w milk in 1X PBS-Tween 20 (0.05%) overnight at 4°C. Subsequently, the plates were incubated with the plasma of the subjects with serial dilutions ranging from 1/250 to 1/512,000 (S1 and N proteins), from 1/250 to 1/16,000 (M protein), from 1/200 to 1/12,800 (E protein) and 1/10 to 1/16,000 (NSP8 protein) for 1 h at 37°C (milk diluted to 1% m/w in PBS 1X-Tween 20 (0.05%). Also, a WHO standard curve (NIBSC code: 20/268) was included from dilutions of 1/40 to 1/2560 for S1, N, and M proteins. Then, plates were incubated with anti-human IgG-HRP (BD, #555788) for 30 minutes at room temperature in the dark. Finally, plates were developed using commercial TMB (BD OptEIA, #555214), which was incubated at room temperature for 15 min in the dark. This reaction was stopped using 2N H_2SO_4, and absorbance was read at optical density 450 nm (OD_{450nm}). To determine the cut-off threshold and establish seropositivity, the average of each dilution factor was obtained for all subjects at the pre-immune visit, and then the average was multiplied by 2.1[20]. Next, the antibody titer was determined for proteins S1, N, M, E, and NSP8 at the highest dilution factor where the corrected OD value was greater than the cutoff value for each dilution factor. Finally, the data were analyzed using the logarithmically transformed concentration. Data for each visit are presented as geometric mean titers (GMT)

in the case of antibody titers, and as geometric mean units (GMU) in the case of interpolated BAU values[20].

To determine the neutralizing capacity of the antibodies, three different assays were performed: surrogate virus neutralization test (sVNT), pseudotyped virus neutralization test (pVNT), and conventional virus neutralization test (cVNT). For the sVNT assay, a Genscript commercial kit (Cat# L00847-A) was used to evaluate the neutralizing capacity of antibodies against the S1-RBD of the S protein of SARS-CoV-2 of WT and Omicron B.1.1.529 (Cat# Z03730) strains, as previously described[18,21]. The neutralizing antibody titer corresponding to each sample was determined by the highest dilution that showed a percentage inhibition greater than or equal to 30%. Samples with a percentage inhibition≤30% at the lowest dilution (1:4) were assigned as seronegative with a titer of 2. Samples were considered seropositive when their titers were higher than the pre-immune titer[18,21]. The pVNT was performed using an HIV-1 virus expressing firefly luciferase as a reporter gene and SARS-CoV-2 pseudotyped S-glycoproteins (HIV-1-SΔ19) of the B.1 (D614G) and Omicron lineage, as previously described[18]. Briefly, plasma samples were serially diluted in DMEM from 1:3 to 1:8.748 and mixed with approximately 4.5 ng of HIV-1-SΔ19 p24 equivalents in a plate. The estimate of ID_{80} (80% inhibitory dilutions) was obtained by a 4-parameter nonlinear regression curve fit, measured as percentage neutralization determined by the difference in mean relative light units (RLU) between test samples and pseudotyped virus controls. Finally, for cVNT, assays were performed by the reduction of cytopathic effect (CPE) in Vero E6 cells (ATCC CRL-1586) seeded in 96-well plates (4×10^4 cells/well)[18,21]. Briefly, the cells were infected with a SARS-CoV-2 strain obtained by viral isolation in tissue culture (strain 33782CL-SARS-CoV-2, variant D614G). A total of 100 µl of 33782CL-

SARS-CoV-2 (at a dose of 100 $TCID_{50}$) was incubated with serial dilutions of the heat-inactivated plasma samples from the volunteers at 1:2 dilution, starting from 1:4 to 1:512 for 1h at 37°C. After 7 days of incubation, the cytopathic effect on the cells was analyzed. The titer of neutralizing antibodies was defined as the highest dilution of plasma that neutralized virus infection, in which CPE was absent. In addition, seropositivity was estimated, considering an increase in the neutralizing antibodies titers of each visit compared to the pre-immune condition. On the other hand, seroconversion was thought to be when the titer of neutralizing results increased 4 times to the pre-immune condition[18].

Activation of $CD4^+$ and $CD8^+$ T cells populations using MPs of SARS-CoV-2

Cells were cultured and ($5x10^5$ per well in 50 µL of media) stimulated with 50 µL of media containing each stimulus for 24 h. Subsequently, cells were stained with six antibodies (Supplementary Table 1), and then incubation was performed for 45 min at 4°C using the stimuli indicated above[18]. Cells were washed twice with 200 µl of PEB buffer, fixed with PFA 2%, and then delivered to the central flow cytometry facility for acquisition on an LSR Fortessa X-20 flow cytometer in the flow cytometry facility at the Pontificia Universidad Católica de Chile.

Supplementary Figures

Supplementary Table 1: Antibodies for flow cytometry

Marker	Fluorophore	Supplier	Dilution
**CD3**	AF700	BioLegend	1:50
**CD4**	BV605	BioLegend	1:50
**CD8**	BV650	BioLegend	1:50
**CD69**	PE	BioLegend	1:50
**OX40**	PE-Cy7	BioLegend	1:50
**CD137**	APC	BioLegend	1:50
**Fixable viability dye**	BV510	BioLegend	1:1000

Supplementary Table 2: Demographics and clinical features of breakthrough cases of children and adolescents vaccinated with CoronaVac®.

Subject	Age range	Sex	Comorbidities
1	3-5 yo	F	Asthma
2	3-5 yo	M	Allergic rhinitis
3	6-11 yo	F	Precocious puberty
4	6-11 yo	F	Not present
5	6-11 yo	F	Not present
6	6-11 yo	M	Not present
7	6-11 yo	M	Allergic rhinitis
8	6-11 yo	M	Not present
9	6-11 yo	M	Mood disorder, allergic rhinitis, obesity.
10	12-17 yo	F	Anxiety and hypermenorrhea
11	12-17 yo	F	Not present
12	12-17 yo	F	Not present

F: female; M: male; yo: years-old.

Supplementary Table 3: Seropositivity rates, seroconversion rates and Geometric Mean Titers (GMT) of circulating neutralizing antibodies against SARS-CoV-2 RBD WT and Omicron variant.

Time	Indicators	WT	Omicron
**2nd dose + 4 weeks**	Seropositivity	12/12	0/12
	%	100	0
	Seroconversion	12/12	0/12
	%	100	0
	GMT	64	2.1
	95% CI	2.0-2-0	1.9-2.4
**2-8 weeks post-infection**	Seropositivity	12/12	12/12
	%	100	100
	Seroconversion	12/12	10/12
	%	100	83.3
	GMT	406.4	30.1
	95% CI	225.3-651.9	19.3-75.1

GMT: Geometric mean titer; WT: Wild-type; CI: Confidence interval.

Supplementary Table 4: Seropositivity rates, seroconversion rates, and Geometric Mean Titers (GMT) of circulating neutralizing antibodies against SARS-COV-2 RBD.

Antibody IgG	Indicators	S protein	N protein	M protein	E protein	NSP8 protein
**2nd dose + 4 weeks**	Seropositivity	12/12	11/12	4/12	0/12	4/12
	%	100	91.7	33.3	0	33.3
	Seroconversion	12/12	10/12	3/12	0/12	4/12
	%	100	83.3	25	0	33.3
	GMT	2520	1888	264.9	112.2	7.8
	95% CI	1788-3550	824.5-4322	123.6-567.4	87.1-144.7	1.3-2.0
**2-8 weeks post-infection**	Seropositivity	12/12	12/12	12/12	0/12	3/12
	%	100	100	100	0	25.0
	Seroconversion	12/12	12/12	12/12	0/12	3/12
	%	100	100	100	0	25.0
	GMT	42715	60408	7127	117.3	31.8
	95% CI	28733-63501	30332-120306	4079-12454	82.8-166.3	45.4-500.5

Supplementary Figure 1:

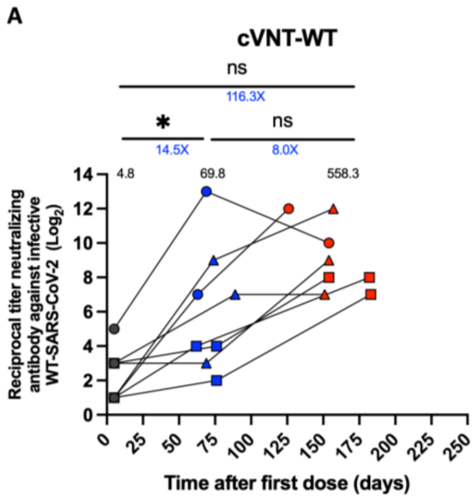

Supplementary Figure 1: Titers of neutralizing antibodies by conventional virus neutralization assay (cVNT) against SARS-CoV-2 in plasma of breakthrough cases of children and adolescents vaccinated with CoronaVac®. A) Neutralizing antibodies titers evaluated by cVNT with $TCID_{50}$ for the WT SARS-CoV-2, expressed as GMTs in twelve pediatric volunteers. The black, blue, and red colors represent the samples evaluated at pre-immune, 2nd dose + 4 weeks, and 2 to 8 weeks post-infection, respectively, by the before-after graph. Circles, triangles and squares correspond to subjects 3-5 yo, 6-11yo and 12-17 yo, respectively. The values on each column indicate the geometric mean. The values below the significance line indicate the times of change between the corresponding geometric means (Red: decrease, Blue: increase). Transformed data, represented as reciprocal dilution in logarithm base 2 on a linear scale. The data were analyzed using a mixed effects model, ***p<0.001, **p<0.001; *p<0.05, not significant (ns).

Supplementary Figure 2:

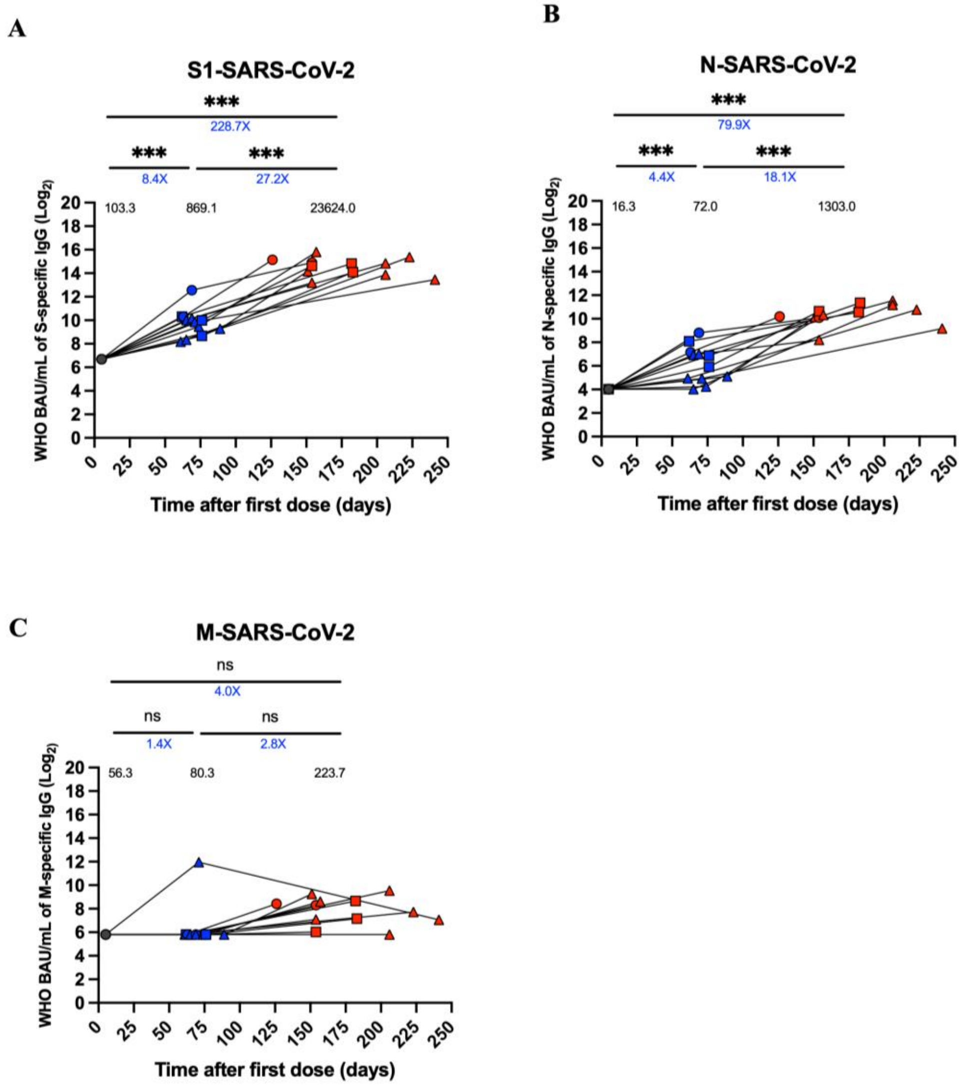

Supplementary Figure 2: Levels of specific IgG antibodies against S1, N, and M SARS-CoV-2 proteins in the plasma of breakthrough cases in BAU/mL. A) Antibody titer in BAU/mL against S1 protein. B) Antibody titer against N protein. C) Antibody titer against M protein. The black, blue, and red colors represent the samples evaluated at pre- immune, 2nd dose + 4 weeks, and 2 to 8 weeks post-infection, respectively, by the before-after graph. Circles, triangles and squares correspond to subjects 3-5 yo, 6-11 yo and 12-17 yo, respectively. An indirect ELISA assay performed *in-house* on plasma from twelve breakthrough cases was used. Values on the significance line indicate an increase in GMU of the two compared time points, and red values on the significance line indicate a decrease in GMU of the two compared time points. The data were analyzed using a mixed effects model, ***p<0.001, **p<0.001; *p<0.05, not significant (ns).

Supplementary figure 3

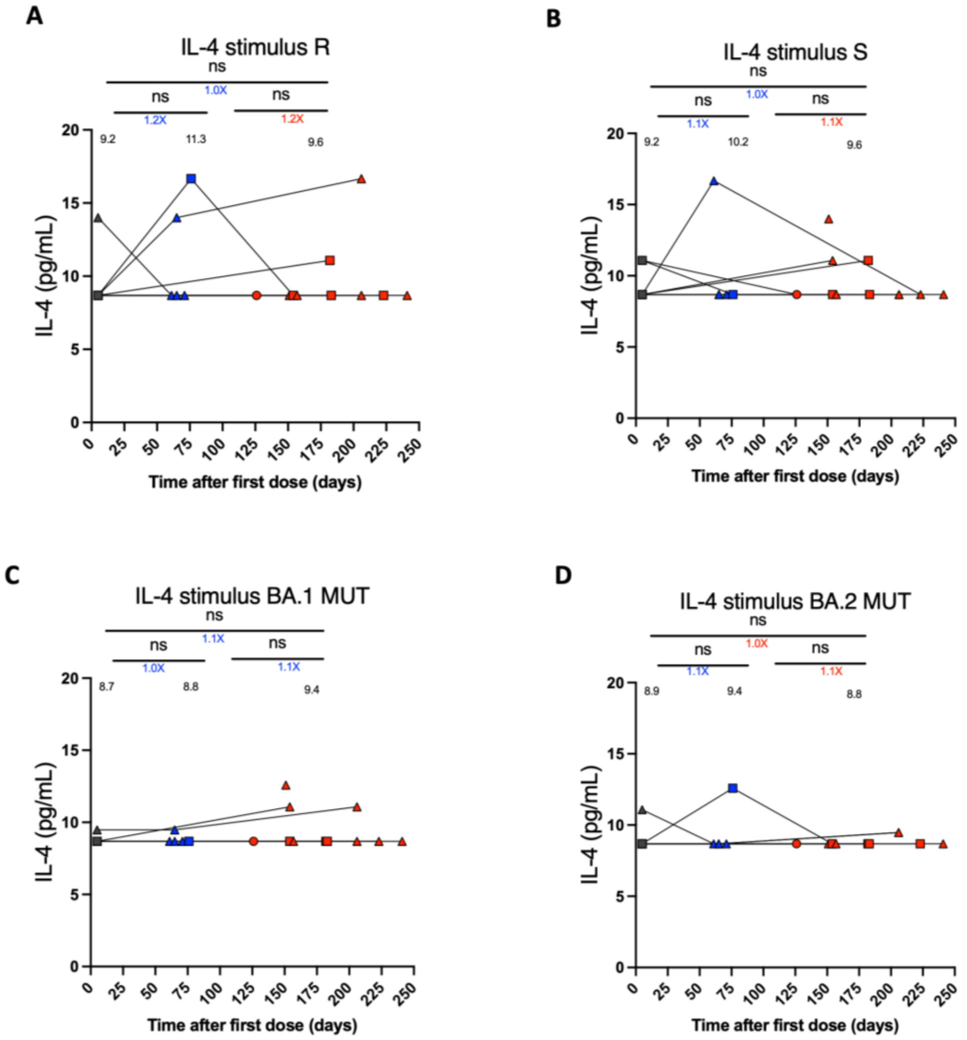

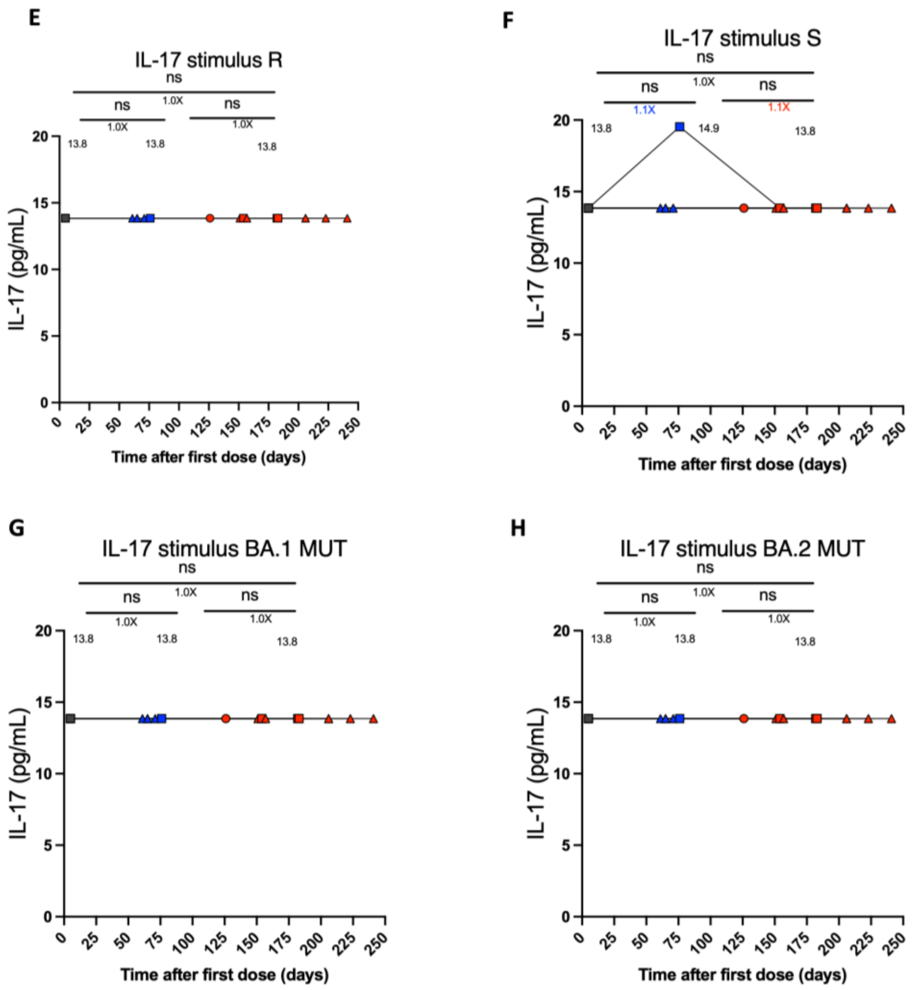

Supplementary Figure 3: IL-4 and IL-17 secretion by PBMCs stimulated using MPs of SARS-CoV-2 of breakthrough cases of children and adolescents vaccinated with CoronaVac®. IL-4 and IL-17 secretion was quantified in supernatants of PBMCs of twelve breakthrough cases upon stimulation with MPs derived from SARS-CoV-2 proteins by Multiplex assay. A-D) IL-4 secretion (pg/ml) by PBMCs stimulated with MP-R and MP-S of WT SARS-CoV-2, and MP-BA.1 and BA.2 of protein S of the Omicron variant of SARS-CoV-2, respectively. E-H) IL-17 secretion (pg/ml). The black, blue, and red colors represent the samples evaluated at pre-immune, 2nd dose + 4 weeks, and 2 to 8 weeks post-infection, respectively, by the before-after graph. Circles, triangles and squares correspond to subjects 3-5 yo, 6-11 yo and 12-17 yo, respectively. The values below the significance line indicate the times of change between the corresponding media (Red: decrease, Blue: increase). The data were analyzed using a mixed effects model, ***p<0.001; **p<0.01; *p<0.05, not significant (ns).
